# Compound heterozygosity for a novel and a recurrent *MFRP* gene mutation in a family with the nanophthalmos-retinitis pigmentosa complex

**Published:** 2009-09-05

**Authors:** Juan Carlos Zenteno, Beatriz Buentello-Volante, Miguel A. Quiroz-González, Miguel A. Quiroz-Reyes

**Affiliations:** 1 Department of Biochemistry, Faculty of Medicine, National Autonomous University of Mexico, Mexico City, Mexico; 2 Department of Genetics-Research Unit, Institute of Ophthalmology, Conde de Valenciana, Mexico City, Mexico; 3 Department of Cornea, Institute of Ophthalmology, Conde de Valenciana, Mexico City, Mexico; 4 Department of Retina, Institute of Ophthalmology, Conde de Valenciana, Mexico City, Mexico

## Abstract

**Purpose:**

To report a new familial case of the recently described autosomal recessive syndrome of nanophthalmos-retinitis pigmentosa-foveoschisis-optic disc drusen, which arises from compound heterozygosity for Membrane Frizzled-Related Protein (*MFRP*) mutations in a sibling pair of Mexican origin.

**Methods:**

Ophthalmological assessment included slit-lamp and dilated fundus examination, applanation tonometry, fundus photography, A-mode and B-mode ultrasound examination, electroretinogram, fluorescein retinal angiography, optical coherence tomography, and electrooculogram in both affected siblings. Molecular genetic analysis consisted of PCR amplification and direct automated sequence of the complete coding region of the *MFRP* gene. In addition, allele-specific cloning and sequencing techniques were used to characterize a heterozygous *MFRP* frameshift mutation.

**Results:**

Clinical examination revealed high hyperopia of > +16 diopters while electroretinographic and fluorangiographic studies demonstrated a retinal dystrophy compatible with retinitis pigmentosa. Ultrasound examination showed nanophthalmos (eye axial length <15 mm) and optic disc drusen while optical coherence tomography evidenced cystoid macular edema. Nucleotide sequencing in DNA from both affected siblings disclosed the presence of two *MFRP* mutations: a novel heterozygous point mutation predicting a nonsense change from tyrosine (TAC) to a stop signal (TAA) at codon 317, and a heterozygous 1 bp deletion in exon 5, predicting a prematurely truncated protein (p.Asn167Thr*fs*X25).

**Discussion:**

The third known family with the syndrome of nanophthalmos-retinitis pigmentosa-foveoschisis-optic disc drusen is presented. This is the first demonstration of compound heterozygosity for *MFRP* mutations as the source of the disease. The affected siblings described here are the youngest patients with the disease reported to date and the comparison of their clinical data with previous individuals with this syndrome suggest that some aspects of the phenotype are probably age-dependent.

## Introduction

The complex of nanophthalmos-retinitis pigmentosa-foveoschisis-optic disc drusen is a distinct autosomal recessive entity recently identified in two unrelated families from Mexico and Spain [1,2]. The complex is caused by homozygous truncating mutations in Membrane Frizzled-Related Protein (*MFRP*), a gene located at chromosome 11q13 that encodes a membrane receptor protein highly expressed in retinal tissue which plays a potential role in the WNT pathway [3]. The two described families have distinct homozygous mutations, yet both have presented with a strikingly similar phenotype characteristically affecting structures located at the posterior pole of the eye [2]. Nanophthalmos, an anomaly in which eyes have a short axial length (13–18.5 mm), causes high hyperopia, typically ranging between +8.00 to +25.00 diopters [4]. Secondary glaucoma due to shallow anterior chamber is a frequent finding in patients with this condition [5-7].

This syndrome has emerged as a novel form of syndromic retinitis pigmentosa with progressive photoreceptor dysfunction initiating typically at the second or third decade of life. Both familial cases currently known carry homozygous frameshift mutations at *MFRP* exon 5, predicting the introduction of premature stop codons [1,2]. Previously, *MFRP* mutations were identified in subjects with nanophthalmos and high hyperopia but without retinal dysfunction [8]. It is still not known why some *MFRP* mutations cause isolated nanophthalmos, while others trigger the nanophthalmos-retinitis pigmentosa-foveoschisis-optic disc drusen complex.

In this work, we describe the clinical and genetic features of a new familial case of the syndrome of nanophthalmos-retinitis pigmentosa-foveoschisis-optic disc drusen in which two distinct *MFRP* mutations, including a novel nonsense change, were demonstrated. The study participants presented here are the youngest reported to date with the syndrome. Our study findings suggest that some aspects of the phenotype are probably age-dependant. This is the first demonstration of compound heterozygosity as the source of this unusual syndromic form of microphthalmos and retinitis pigmentosa.

## Methods

### Clinical studies: Two sibs were studied

Ophthalmological examinations included best-corrected visual acuity (BCVA), slit-lamp and dilated fundus examination, applanation tonometry, fundus photography, A-mode and B-mode ultrasound examination (US), electroretinogram (ERG), fluorescein retinal angiography (FA), optical coherence tomography (OCT), and electrooculogram. Affected participants also underwent systemic evaluation (not only ocular examination, but also complete physical examination was performed) by a geneticist. The patients were recruited from the Department of Retina of our institution (Institute of Ophthalmology “Conde de Valenciana”) and belonged to a Mexican Mestizo family from the central region of the country. No consanguinity was recorded among their parents, who had no vision abnormalities. The patients were the only two siblings from this marriage.

### Molecular genetic analyses

The two affected siblings participated in the study after providing informed consent. The study protocol was approved by the Institutional Review Board. Genomic DNA was obtained in both participants from peripheral blood lymphocytes according to standard methods [9]. The entire coding sequences and exon-intron boundaries of *MFRP* (located at 11q23, 13 exons) were amplified by PCR using pairs of primers derived from the normal published sequences (Ensembl sequence ENST00000261980). Primer sequences and annealing temperatures for PCR are shown in Table 1. Direct sequencing of PCR amplified products was performed using the Big Dye Terminator Cycle Sequencing kit (Applied Biosystems, Foster City, CA), with about 10 ng of template DNA added to each reaction. Samples were run in an ABI Prism 310 Genetic Analyzer (Applied Biosystems). Sequences were compared manually. Additionally, an allele-specific cloning and sequencing approach was used to precisely characterize a *MFRP* frameshift mutation. Briefly, new *MFRP* exon 5 products were amplified from genomic DNA, gel-purified, ligated by means of a TA-ligation method into the TA-cloning vector pGEM-T (Promega, Madison, WI), and subcloned into DH5α *E. coli* competent cells (Invitrogen, Carlsbad, CA) as previously described [10]. The plasmid inserts were sequenced by following the aforedescribed protocol and by using the forward pUC/M13 primer.

**Table 1:** PCR primers for *MFRP* gene amplification

**Exon**	**Oligonucleotide sequence (5′-3′)**	**Annealing temperature (°C)**	**PCR product size (bp)**
1–4 F	GGTCTTGGGCTGTCACAGG	60.3	843
1–4 R	CCACCCCGTCATCTTGGGC		
2 R	CCTTCTGTTGGGTATTCCTC	(for sequencing only)	
5 F	CTTGAGAATAAAGGACCTCA	59.5	327
5 R	CTTTAGATAGTGGTTCAGGA		
6 F	ATAGGACTGCAAGGCCCAGG	58.7	287
6 R	CCTTTGACAGGACTGGGAGT		
7–8 F	ACTCCCAGTCCTGTCAAAGG	59.2	582
7–8 R	TTCCCATTACACTAACTTGG		
9 F	TTAAGAACCACCAATGATG	54.6	370
9 R	TGGAGAATGGAATGTGCTGG		
10 F	AGCTCAGAGCCAGGCCTGT	58.2	264
10 R	CCTGGAGGTGCCTCTACT		
11 F	GGACAGACAAGGGCTCTGGA	59.1	304
11R	ACTGTGCAGTACGGCAGTAGG		
12 F	ATTCGGTGACTTGCCACAGG	59.1	581
12 R	TTGTTCCCCTGCGTGCCAGC		

## Results

### Patient #1

**Figure 1:**
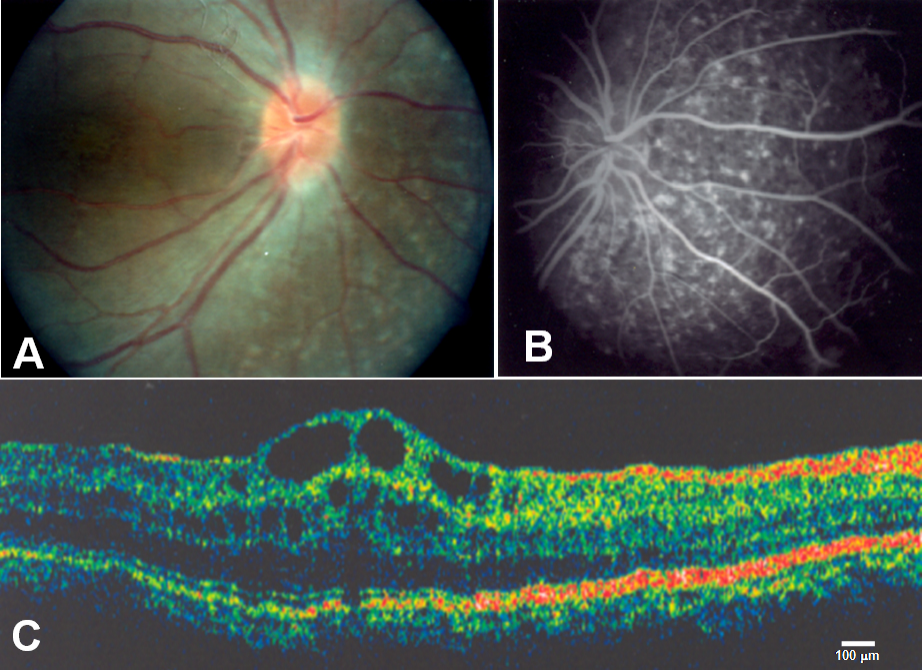
Eye phenotype in Retinitis pigmentosa-nanophthalmos complex. **A**: Fundus photograph of the right eye from patient #1 reveals optic disc drusen, diffuse retinal pigment epithelium atrophy, and blunting of the macular reflex. **B**: Fluorescein angiography shows choroidal transmission hyperfluorescence corresponding to retinal pigment epithelium atrophy (patient #1, right eye). **C**: OCT image demonstrates cystoid macular edema, inner retinal layers splitting with discrete bridging elements at the fovea, and macular cysts (patient #2, left eye).

An 18-year-old girl, patient #1 had a BCVA of 20/200 and refractive error of +16.75 OU. In both eyes, her intraocular pressure was 12 mmHg, normal horizontal corneal diameters (11.5 mm), and no anterior chamber angle anomalies. This patient had progressive nyctalopia and decreasing visual acuity since infancy. Funduscopic examination disclosed very small optic cups (<20%), cystic lesions on macular areas, diminished foveal reflex in both eyes, and retinal pigmented epithelium (RPE) mottling and atrophy mainly at posterior pole and peripheral retina in both eyes (Figure 1A). There was no clinical evidence of retinoschisis. FA showed transmission defects corresponding to the observed mottled areas of RPE atrophy (Figure 1B). ERG showed abolished scotopic light response in both eyes while photopic response was recordable but subnormal OU. The Arden ratio was 144% OD and 108% OS (normal values: 180%–200%). Ultrasonographic examination demonstrated eye axial length of 14.79 mm (OD) and 14.65 mm (OS), but it showed optic nerve drusen and thickened choroid in both eyes. OCT imaging revealed increased foveal thickness that was 331 μm OD and 317 μm OS. OCT also showed hyporeflective images of cystic appearance, and splitting of inner retinal layers.

### Patient #2

The 16 year-old brother of patient #1, patient #2 presented with a BCVA of 20/200 OD and 20/100 OS. In both eyes, he had a refractive error of +16.75 OU, intraocular pressure of 12 mmHg, normal horizontal corneal diameters (11.5 mm), and no anterior chamber angle anomalies. Like his sister, this patient had progressive nyctalopia and decreasing visual acuity since infancy. He had also cystic lesions on macular areas, diminished foveal reflexes, and RPE mottling and atrophy mainly at posterior pole and peripheral retina in both eyes. Also in both eyes, he had cystic lesions on macular area, diminished foveal reflex, and RPE mottling and atrophy mainly at posterior pole and peripheral retina. There was no clinical evidence of retinoschisis. FA showed diffuse transmission defects corresponding to the observed areas of RPE mottling and atrophy. ERG demonstrated a subnormal scotopic light response in OD and an abolished response in OS. OCT imaging revealed this patient had increased foveal thickness of 399 μm OD and 409 μm OS, as well as hyporeflective images of cystic appearance, and apparent splitting of inner retinal layers (Figure 1C). The Arden ratio was 122% OD and 116% OS. Ultrasonography revealed his eye axial length was 14.93 mm OD, and 14.67 mm OS. He had optic nerve drusen and thickened choroids in both eyes.

### Molecular genetic findings

**Figure 2:**
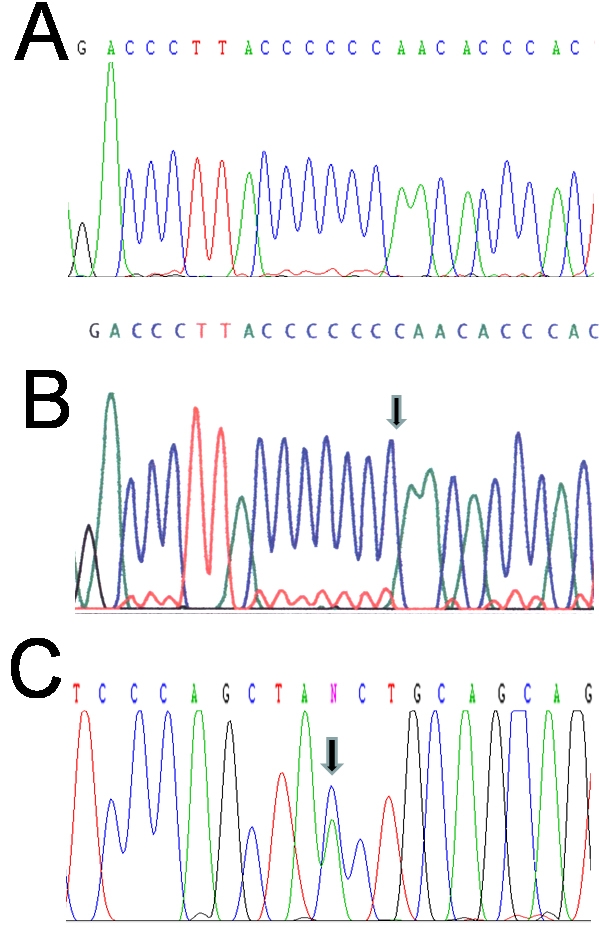
*MFRP* gene mutations in Retinitis pigmentosa-nanophthalmos complex. **A**: Partial nucleotidic sequence of *MFRP* exon 5 in DNA from patient # 1 shows a heterozygous 1 bp deletion (c.498delC), which predicts a prematurely truncated protein (p.Asn167Thr*fs*X25). Compare this illustration with the normal sequence shown in **B** in which arrow points to the deleted nucleotide. **C**: Partial DNA sequence of *MFRP* exon 8 shows a heterozygous point mutation from C to A (arrow), predicting a nonsense change from tyrosine (TAC) to a stop signal (TAA), at codon 317.

Nucleotide sequence in DNA from both affected siblings disclosed the presence of two *MFRP* mutations: a heterozygous 1-bp deletion (c.498delC) in exon 5 (Figure 2A,B), predicting a prematurely truncated protein (p.Asn167Thr*fs*X25); and a heterozygous point mutation from C to A, predicting a nonsense change from Tyrosine (TAC) to a stop signal (TAA), at codon 317 in exon 8 (Figure 2C). This nonsense mutation has not been previously described while the c.498delC mutation was recently identified by Crespi et al. [2] in a familial case in Spain of the nanophthalmos-retinitis pigmentosa syndrome.

## Discussion

Nanophthalmos is an ocular anomaly characterized by the combination of microphthalmos, microcornea, and a tendency toward spontaneous or postsurgical uveal effusions [11,12]. Nanophthalmos is sometimes referred to as “simple microphthalmos” [13,14]. The short distance between the lens and retina in nanophthalmic eyes causes extreme hyperopia with refractive defects ranging between +8.00 to +25.00 diopters. Typically, nanophthalmic eyes have an axial length of 13–18.5 mm and are associated with a shallow anterior chamber and thickening of both the choroidal vascular bed and sclera [15-17].

In 2006, Ayala-Ramirez et al. [1] described the ophthalmologic features of a Mexican family in whom four siblings were affected by a novel autosomal recessive eye disease characterized by posterior microphthalmos, retinitis pigmentosa, foveoschisis, and optic disc drusen. As human *MFRP* mutations result in nanophthalmos and mutations in the orthologous *Mfrp* gene causes the recessive mouse retinal degeneration *rd6*, these authors searched for *MFRP* mutations as a source for this novel phenotype. A homozygous frameshift P166fsX199 mutation in MFRP segregated with the disease in that pedigree, validating mutations at this gene as the responsible for the phenotype [1].

The panocular phenotype observed in patients with the nanophthalmos-retinitis pigmentosa complex (microphthalmia, retinitis pigmentosa, foveoschisis, and optic disc drusen) suggests that *MFRP* plays a wide role in eye development, functioning both as a regulator of axial eye length and as a critical molecule for photoreceptor function. Recent studies in the Mfrprd6/rd6 mouse model indicate that MFRP is necessary for photoreceptor maintenance [18].

The affected siblings presented in this work are the youngest described to date. Several clinical features are important to note. Fundus examination in both patients revealed multiple, irregular, round yellow-white flecks, mainly located at the posterior pole retina and did not demonstrate the presence of “bone spicule” hyperpigmentation. The absence of this hyperpigmentation is significant because previous reports documented its presence in all patients (seven from two unrelated families) with nanophthalmos-retinitis pigmentosa complex. This is an interesting finding as mice homozygous for *rd6* have small, evenly spaced white dots throughout their retinas that become apparent by eight weeks of age. Homozygous *rd6* mice begin to show clinical signs of retinal degeneration at seven months of age. By 15 months, the fundus has developed a mildly pigmented granular and mottled appearance; individual spots can still be seen, although less frequently, in mice up to about two years old [19]. These data, along with our observations, suggest that *MFRP* mutations in both mice and humans lead to a fundus phenotype characteristic of “typical” retinitis pigmentosa, with retinal pigmentary changes occurring in the form of fine mottling or granularity with surrounding areas of atrophy in the earlier stages followed by the typical “bone-spicule” pattern of hyperpigmentation in midperipheral retina, at later stages.

Each participant from the original family [1] exhibited localized foveal schisis with no clinical, fluorangiographic, or OCT evidence of cystoid macular edema or macular cysts. Retinal OCT in patients from the family described by Crespi et al. [2] and in the young siblings we describe here showed foveal cystic lesions reminiscent of cystoid macular edema. There was, however, schisis of external retinal layers evident in the present study’s siblings, suggesting the occurrence of two independent maculopathies: foveoschisis and cystoid macular edema. Although this finding suggests that it is probable that macular cysts leads to foveoschisis, it is also likely that the combined effect of both nanophthalmos and macular cysts could account for the localized foveal schisis characteristic of this syndromic form of retinitis pigmentosa. It is also possible that foveoschisis is a permanent sequela after cystoid edema resolves in nanophthalmic eyes, contrary to that which occurs in normal sized eyes. Cystoid macular edema is a frequent finding in retinitis pigmentosa and, according to OCT results, seems to occur in 32%–49% of these patients [20,21]. Glaucoma is another finding that suggests there is an age-dependent component to nanophthalmos-retinitis pigmentosa complex. While not observed in young or middle-aged patients in this study and the one by Ayala-Ramirez et al. [1], glaucoma was noted in older participants in the research performed by Crespi et al. [2]. Further study of additional subjects of a range of ages with this complex would help to clarify these issues.

Finally, this case of *MFRP* compound heterozygosity suggests that the disorder, or at least the carrier status, could be more frequent than previously thought. We propose to suspect the diagnosis of this complex diseases in those patients with retinitis pigmentosa who also exhibit high refractive errors ranging between +8.00 to +25 diopters.
